# Oxidative balance score inversely associated with the prevalence and incidence of metabolic syndrome: analysis of two studies of the Korean population

**DOI:** 10.3389/fnut.2023.1226107

**Published:** 2023-08-16

**Authors:** Hye-Min Park, Tea-Hwa Han, Yu-Jin Kwon, Jun-Hyuk Lee

**Affiliations:** ^1^Primary Care Research Center, National Health Insurance Service Ilsan Hospital, Goyang, Republic of Korea; ^2^Health-IT Center, Yonsei University Severance Hospital, Seoul, Republic of Korea; ^3^Department of Family Medicine, Yongin Severance Hospital, Yonsei University College of Medicine, Yongin, Republic of Korea; ^4^Department of Family Medicine, Nowon Eulji Medical Center, Eulji University School of Medicine, Seoul, Republic of Korea; ^5^Department of Medicine, Hanyang University School of Medicine, Seoul, Republic of Korea

**Keywords:** oxidative balance score, pro-oxidant, antioxidant, metabolic syndrome, Korean Genome and Epidemiology Study

## Abstract

**Background:**

Pro-oxidant/antioxidant imbalances leading to chronic inflammation and insulin resistance can contribute to the development of metabolic syndrome (MetS). Oxidative Balance Score (OBS), a comprehensive measure of exposure to pro- and anti-oxidants, represents an individual’s total oxidative balance. This study aimed to evaluate the association between OBS and MetS using two large datasets.

**Methods:**

We analyzed data from 2,735 adults older than 19 years from the 2021 Korean National Health and Nutritional Examination Survey (KNHANES) and 5,807 adults aged 40–69 years from the Korean Genome and Epidemiology Study (KoGES). In each dataset, OBS was categorized into sex-specific tertiles (T).

**Results:**

In KNHANES, the odds ratios and 95% confidence intervals for prevalent MetS in T3, compared to T1, were 0.44 (0.29–0.65) in men and 0.34 (0.23–0.50) in women after adjusting for confounders. In KoGES, the hazard ratios and 95% confidence intervals for incident MetS in T3, compared to T1, were 0.56 (0.48–0.65) in men and 0.63 (0.55–0.73) in women after adjusting for confounders.

**Conclusion:**

OBS appears to be inversely related to MetS, which suggests that adopting lifestyle behaviors that decrease oxidative stress could be an important preventive strategy for MetS.

## 1. Introduction

Metabolic syndrome (MetS) is a cluster of metabolic dysregulations that place individuals at higher risk of type 2 diabetes and cardiovascular disease (1). The global prevalence of MetS has been estimated to be around 25% (2) and is continuously increasing in parallel with the prevalences of obesity and type 2 diabetes (3). According to a meta-analysis of global data, the prevalence of MetS is higher in the Eastern Mediterranean Region (32.9, 95% CI: 28.7–37.2) and the Americas (26.0, 95% CI: 22.7–29.4) (4). One report indicates that the prevalences of MetS in men and women in Korea in 2018 were 27.9 and 17.9%, respectively (5). Additional research suggests that people with MetS spend 1.6 times more on health care than those without MetS ($5,732 vs. $3,581) (6). Accordingly, public health experts should devote more attention to reducing the disease burden of MetS because it carries higher risks of cardiovascular mortality, all-cause mortality, and comorbidities, such as cerebrovascular disease, peripheral vascular disease, and cardiovascular disease, in addition to increased costs (7).

Both insulin resistance and chronic systemic inflammation are key factors in MetS (8). Emerging evidence demonstrates that systemic oxidant stress brought on by insulin resistance and excessive fatty acids activates a reciprocal interaction of downstream inflammatory pathways (9). As such, interest in means with which to control oxidative stress and chronic inflammation in order to mitigate the severity of comorbid chronic diseases and to prevent MetS is growing (10), and several studies have suggested that increasing consumption of dietary antioxidants and decreasing pro-inflammatory dietary behaviors can reduce oxidative stress levels and MetS incidence (11, 12).

In the literature, researchers have highlighted differences in onset and progression of MetS between men and women. These differences can be attributed to various factors, including hormones, adipose tissue distribution, genetics, and lifestyle factors (13). Oxidative balance score (OBS) is a useful tool for assessing an individual’s oxidation–reduction balance, including dietary and lifestyle factors (14). Previous studies have suggested that combined measure of various pro-oxidants and anti-oxidants, including dietary and non-dietary factors, are closely associated with metabolic diseases (14–16). OBS could provide more comprehensive evaluation of oxidative stress and antioxidant capacity than individual markers alone. However, lack of standardized methodology for calculating OBS makes it difficult to compare scores across different populations or studies. Also, there is limited clinical utility for predicting disease risk (14, 17).

Although several studies have reported a relationship between OBS and MetS, results are inconsistent. A cross-sectional study conducted in Korea reported that OBS is inversely related to the risk of MetS in adults aged ≥40 years (18), whereas an Iranian cross-sectional study did not show a significant association between OBS and MetS components (19). Moreover, there is limited evidence of the association between OBS and the incidence of MetS from prospective cohort studies. Our study aimed to address a gap in current knowledge by investigating the association between OBS and MetS prevalence and incidence in two large, independent population-based datasets in a sex-specific manner.

## 2. Materials and methods

### 2.1. Study population

This study utilized two population-based cohorts: the 2021 Korea National Health and Nutrition Examination Survey (KNHANES) and the Korean Genome and Epidemiology Study (KoGES). The KNHANES is a nationwide representative population-based survey. The cohort profile was described in detail in a previous study (20). The 2021 KNHANES dataset included 7,090 participants aged 19 years and older. The KoGES Ansan and Ansung study is a community-based prospective cohort study. The study design and procedures have been described in detail in a previous study (21). The KoGES Ansan and Ansung cohort included 10,030 adults aged between 40 and 69 years. This survey was first conducted in 2001 and 2002, and participants were followed up every 2 years. In the present study, we included participants with up to eight follow-up evaluations conducted between 2017 and 2018.

**Figure 1:**
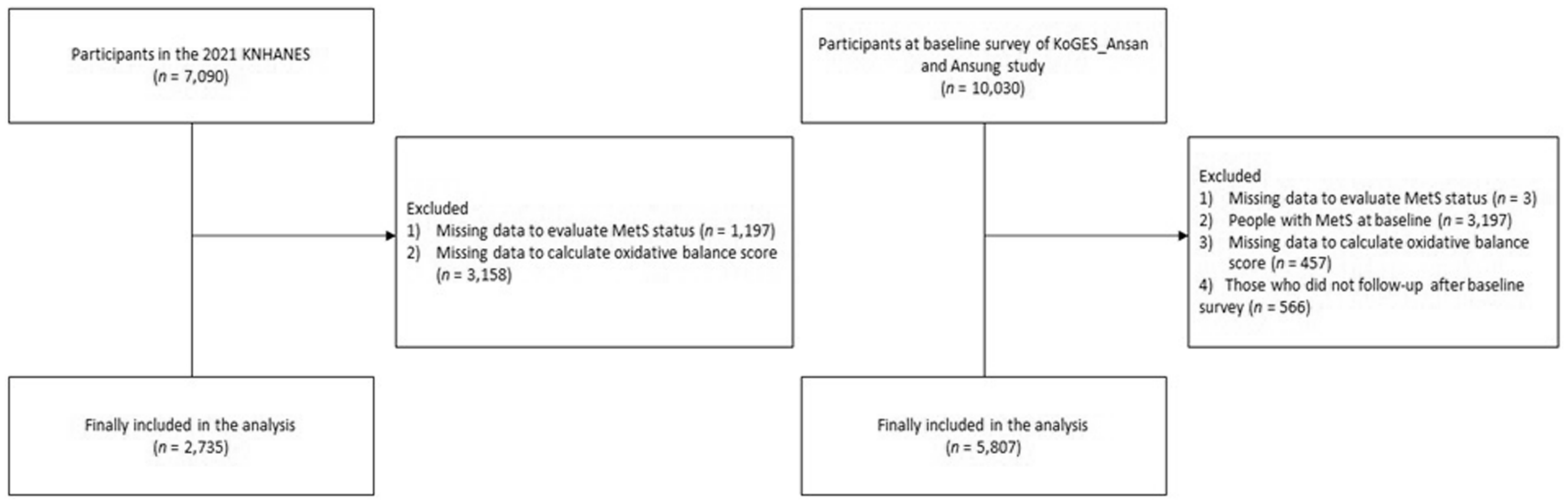
Flow chart of the study population.

A flowchart of the study population is provided in Figure 1 (KNHANES and KoGES). Among the 7,090 participants in the 2021 KNHANES dataset, 2,735 participants (1,185 men and 1,550 women) were finally included for analysis after excluding participants who had missing data with which to evaluate MetS (*n* = 1,197) and to calculate OBS (*n* = 3,158). Among the 10,030 participants in the KoGES dataset, 5,807 participants (2,921 men and 2,886 women) were finally included for analysis after excluding participants with missing data needed for MetS evaluation (*n* = 3), those with MetS at baseline (*n* = 3,197), those who lacked data needed to calculate OBS (*n* = 457), and those who did not participate in a follow-up after the baseline survey (*n* = 566).

All participants in KNHANES and KOGES provided informed consent for data collection. The study protocol conformed to the ethical guidelines of the 1964 Declaration of Helsinki and its later amendments. This study was approved by the Institutional Review Board (IRB) of Nowon Eulji Medical Center (IRB number: 2021-09-025).

### 2.2. Oxidative balance score assessment

OBS was calculated from data on six pro-oxidant factors and five antioxidant factors selected based on previous studies (14, 22–24), and data for all 11 factors were available in the both KHNANES and KoGES datasets. Table 1 presents the OBS assignment scheme. Pro-oxidant factors included saturated fatty acid (SFA), total iron intake, smoking status, drinking status, obesity status, and abdominal obesity status (14, 25, 26). Each query was scored 0, 1, or 2, except for abdominal obesity status, which was scored 0 or 1. The scores for SFA and total iron intake were assigned 0–2 points according to sex-specific tertile values (low, 2; intermediate, 1; high, 0) of each variable. The scores for never smokers, former smokers, and current smokers were 2, 1, and 0, respectively. The scores for non-drinkers, mild-to-moderate drinkers (1–29 g/day in men, 1–19 g/day in women), and heavy drinkers (≥30 g/day in men, ≥20 g/day in women) were 2, 1, and 0, respectively. Obesity received 0 points, overweight received 1 point, and normal weight received 2 points. Zero points were given for abdominal obesity and 1 point for no abdominal obesity. Antioxidant factors included physical activity (high intensity, 2; moderate intensity, 1; low intensity, 0) and intake of omega-3 poly-unsaturated fatty acid (PUFA) to omega-6 PUFA ratio, vitamin C, vitamin E, and beta-carotene, which were each assigned 0–2 points according to sex-specific tertile values (low, 0; intermediate, 1; high, 2) for each variable. Using OBS, which had a possible maximum of 21 points, we divided the individuals into tertile groups by sex. Higher OBS indicate higher antioxidant properties. In the KoGES and KNHANES datasets, OBS cut-off points were stratified into three categories: for men, T1 (≦11 for KoGES and ≦10 for KNHANES), T2 (12–13 for KoGES and 11–12 for KNHANES), and T3 (≧14 for KoGES and ≧13 for KNHANES); for women, T1 (≦12), T2 (13–14), and T3 (≧15) in both datasets.

**Table 1:** Oxidative balance score assignment scheme.

OBS components	Assignment scheme^*^
1. Saturated fatty acid [P]	0 = high (3rd tertile), 1 = intermediate (2nd tertile), 2 = low (1st tertile)
2. Total iron intake [P]	0 = high (3rd tertile), 1 = intermediate (2nd tertile), 2 = low (1st tertile)
3. Smoking status [P]	0 = current smoker, 1 = former smoker, 2 = never smoker
4. Drinking status [P]	0 = heavy drinker (≥30 g/day in men, ≥20 g/day in women), 1 = mild-to-moderate drinker (<30 g/day in men, <20 g/day in women), 2 = non-drinker
5. Overweight/obese [P]	0 = obese, 1 = overweight, 2 = normal
6. Abdominal obesity [P]	0 = abdominal obesity, 1 = normal
7. Omega-3/omega-6 PUFA ratio [A]	0 = low (1st tertile), 1 = intermediate (2nd tertile), 2 = high (3rd tertile)
8. Vitamin C intake [A]	0 = low (1st tertile), 1 = intermediate (2nd tertile), 2 = high (3rd tertile)
9. Vitamin E intake [A]	0 = low (1st tertile), 1 = intermediate (2nd tertile), 2 = high (3rd tertile)
10. Total beta-carotene intake [A]	0 = low (1st tertile), 1 = intermediate (2nd tertile), 2 = high (3rd tertile)
11. Physical activity [A]	0 = low (<7.5 METs-hr/day), 1 = moderate (7.5–30 METs-hr/day), 2 = high (>30 METs-hr/day)

* Low, intermediate, and high categories correspond to sex-specific tertile values among participants in the KoGES at the baseline survey or participants in the 2021 KNHANES. P, pro-oxidant; A, anti-oxidant; PUFA, poly-unsaturated fatty acid; MET, metabolic equivalent of task; KoGES, Korean Genome and Epidemiology Study; KNHANES, Korean National Health and Nutrition Examination Survey.

### 2.3. Definition of MetS

Based on the National Cholesterol Education Program Adult Treatment Panel III definition as amended by the National Heart Lung and Blood Institute and the American Heart Association (27), we defined MetS as the presence of three or more of the following: (1) abdominal obesity as a waist circumference ≥ 90 cm in men and ≥ 85 cm in women based on the 2018 Korean Society for the Study of Obesity guidelines (28); (2) fasting plasma glucose ≥ 5.6 mmol/L or use of oral hypoglycemic medication or insulin; (3) serum triglyceride concentration ≥ 1.7 mmol/L or use of lipid-lowering medication; (4) serum high-density lipoprotein (HDL) cholesterol concentration < 1.0 mmol/L in men or < 1.3 mmol/L in women; and (5) systolic blood pressure (SBP) ≥ 130 mmHg, diastolic blood pressure (DBP) ≥ 85 mmHg, or use of anti-hypertensive medication.

### 2.4. Covariates

Well-trained medical staff members performed the health examination sand health interviews following the KHANES protocol (20) and KoGES protocol, respectively (29). Body mass index (BMI) was calculated as a person’s weight in kilograms divided by the square of their height in meters. Individuals with a BMI of at least 23 kg/m^2^ but less than 25 kg/m^2^ were considered overweight, and those with a BMI of ≥25 kg/m^2^ were considered obese based on the 2018 Korean Society for the Study of Obesity guidelines (28). Abdominal obesity was defined as a waist circumference ≥ 90 cm in men and ≥ 85 cm in women based on the same guidelines (28). Mean blood pressure (MBP, mmHg) was calculated as DBP + 1/3 × (SBP – DBP) (30). Whole blood white blood count (WBC), plasma glucose, serum insulin, total cholesterol, triglycerides, HDL, cholesterol and C-reactive protein (CRP) were assessed using a Hitachi 700–110 Chemistry Analyzer (Hitachi, Ltd., Tokyo, Japan) after at least 8 h of fasting.

Information on smoking, alcohol consumption, physical activity, education level, and household income were obtained from self-reported questionnaires. A never smoker was defined as someone who had never smoked or had smoked fewer than 100 cigarettes in their lifetime. Former smokers were those who had quit smoking and had smoked more than 100 cigarettes during their lifetime. Current smokers were those who were active smokers at the time of the questionnaire and had smoked more than 100 cigarettes during their lifetime.

We further evaluated the daily alcohol intake (g/day) of each participant. Heavy drinkers were men who drank more than 30 g/day and women who drank more than 20 g/day. Mild-to-moderate drinkers were men who drank less than 30 g/day and women who drank less than 20 g/day. Non-drinkers were those who did not drink alcohol.

Physical activity was assessed using the metabolic equivalent of task–hours per week (MET-hrs/day) using the International Physical Activity Questionnaire (31). Participants were classified into low- (<7.5 METs-hr/day), moderate- (7.5–30 METs-hr/day), or high- (>30 METs-hr/day) intensity physical activity groups. An in-person interview concerning nutrition was conducted in the respondent’s home.

Total energy intake and nutritional status were calculated using a validated 112-item food frequency questionnaire (FFQ) developed for KNHANES (32) and 103-item FFQ developed for KoGES (33). Information for each item was collected based on recalls of the average frequency and amount consumed per serving over the past year. From the FFQ, this study considered the total daily intake values of energy (kcal/day), SFA (g/day), total iron (mg/day), omega-3 PUFA to omega-6 PUFA ratio, vitamin C (mg/day), vitamin E (mg/day), and beta-carotene (μg/day).

Participants were classified into elementary/middle school, high school, and college/university education levels. Monthly household income was divided into three groups: <100 million Korean Won, 100–200 million Korean Won, and > 200 million Korean Won.

### 2.5. Statistical analysis

We conducted a normality test, and variables with normal distribution are presented as means ± standard deviations. Serum triglyceride, insulin, and CRP with non-normal distribution are presented as medians (25th percentile, 75th percentile). Continuous variables were compared using a one-way analysis of variance or the Kruskal–Wallis test according to the sex-specific OBS tertiles. All statistical analyses were conducted separately for men and women. Categorical variables are described as numbers (%) and were compared using the chi-square test. The dose–response relationship between OBS and the risk of incident MetS was determined using a Cox proportional hazard spline curve. Kaplan–Meier curves with the log-rank test were used to verify the cumulative incidence of MetS according to the sex-specific OBS tertiles. For KNHANES, we calculated the odds ratio (OR) and 95% confidence intervals (CI) for the prevalence of MetS. For KoGES, we completed univariable and multivariable Cox proportional hazard regression analyses to calculate the HRs and 95% CIs for incident MetS of the second tertile (T2) and highest tertile (T3) groups, compared with the referent lowest tertile (T1) group, in a sex-specific manner. In the fully adjusted model, we adjusted for age, education level, monthly household income, total energy intake, MBP, WBC, fasting plasma glucose, and serum total cholesterol levels based on each population characteristics. Confounders were determined as variables influencing exposure and outcome based on a literature review and univariate analysis (34, 35). We calculated variance inflation factor (VIF) values to assess the degree of multicollinearity among the variables, indicating that a VIF value of five or higher represents a high correlation of the variables (36). In this study, the maximum VIF value observed was 2.1, which corresponded to the variable ‘age’ in women (Supplementary Tables 1, 2). All statistical analyses were performed with SAS statistical software (version 9.4; SAS Institute Inc., Cary, NC, USA) and R software (version 4.1.1; R Foundation for Statistical Computing, Vienna, Austria). *P*-values less than 0.05 were regarded as statistically significant.

## 3. Results

### 3.1. Baseline characteristics of the study population

Table 2 shows the baseline characteristics of the men according to OBS tertile in the KoGES and KNHANES, respectively. In the KoGES, the T3 group had lower fasting glucose, insulin, total cholesterol, triglyceride, and WBC. T3 men had higher intake of SFA, total iron, omega-3 to omega-6 PUFA ratio, vitamin C, vitamin E, and beta-carotene. Since we excluded participants with existing MetS at the baseline survey of KoGES, the proportion of participants with two MetS components was lowest in the T3 group.

**Table 2:** Baseline characteristics of men in the KoGES and 2021 KNHANES.

	Oxidative balance score tertiles							
	Men in KoGES				Men in KNHANES			
Variables	T1 (*n* = 1,128)	T2 (*n* = 977)	T3 (*n* = 816)	*p* ^*^	T1 (*n* = 420)	T2 (*n* = 310)	T3 (*n* = 455)	*p* ^*^
Age, years	51.3 ± 8.7	51.5 ± 9.0	51.5 ± 8.9	0.773	45.9 ± 17.1	50.0 ± 16.8	54.8 ± 17.4	<0.001
MBP, mmHg	95.3 ± 11.6	95.6 ± 12.1	95.1 ± 12.5	0.657	93.2 ± 10.7	91.0 ± 9.9	89.8 ± 10.4	<0.001
Glucose, mg/dL	87.2 ± 17.7	86.1 ± 15.5	85.3 ± 13.6	0.030	104.5 ± 25.3	103.8 ± 19.2	103.1 ± 23.6	0.659
Insulin, IU/	6.9 ± 3.8	6.5 ± 3.3	6.2 ± 3.0	<0.001	10.3 ± 6.7	9.7 ± 7.5	7.8 ± 5.6	<0.001
Total cholesterol, mg/dL	192.0 ± 34.8	192.0 ± 35.9	186.2 ± 34.4	<0.001	191.1 ± 39.1	186.1 ± 39.4	187.8 ± 39.0	0.201
Triglyceride, mg/dL	160.2 ± 99.7	147.4 ± 97.9	134.7 ± 82.9	<0.001	163.6 ± 164.1	142.1 ± 118.5	121.2 ± 75.7	<0.001
HDL cholesterol, mg/dL	45.7 ± 9.7	45.6 ± 9.4	46.2 ± 10.8	0.335	48.2 ± 12.0	47.9 ± 10.0	49.5 ± 11.8	0.087
WBC, 10^9^/μL	6.9 ± 1.8	6.7 ± 1.8	6.4 ± 1.8	<0.001	6.5 ± 1.7	6.3 ± 1.6	5.9 ± 1.5	<0.001
Education level, n (%)				0.858				0.414
Elementary/middle school	475 (42.2%)	397 (40.8%)	329 (40.5%)		61 (14.5%)	44 (14.2%)	85 (18.7%)	
High school	412 (36.6%)	354 (36.4%)	296 (36.4%)		119 (28.3%)	87 (28.1%)	119 (26.2%)	
College/university	239 (21.2%)	221 (22.7%)	188 (23.1%)		240 (57.1%)	179 (57.7%)	251 (55.2%)	
Household income, n (%)				0.267				0.394
<100 million Korean Won	288 (25.7%)	266 (27.5%)	234 (28.7%)		125 (30.0%)	103 (33.3%)	165 (36.4%)	
100–200 million Korean Won	345 (30.8%)	278 (28.7%)	257 (31.6%)		145 (34.8%)	103 (33.3%)	145 (32.0%)	
>200 million Korean Won	487 (43.5%)	425 (43.9%)	323 (39.7%)		147 (35.3%)	103 (33.3%)	143 (31.6%)	
Energy intake, kcal/day	1895.2 ± 593.2	2034.1 ± 680.7	2178.1 ± 749.7	<0.001	2231.6 ± 975.2	2243.5 ± 917.7	2113.0 ± 758.9	0.063
Number of MetS components, n (%)				<0.001				<0.001
0	211 (18.7%)	273 (27.9%)	234 (28.7%)		103 (24.5%)	76 (24.5%)	146 (32.1%)	
1	405 (35.9%)	365 (37.4%)	353 (43.3%)		77 (18.3%)	59 (19.0%)	103 (22.6%)	
2	512 (45.4%)	339 (34.7%)	229 (28.1%)		85 (20.2%)	78 (25.2%)	97 (21.3%)	
≥3	–	–	–		155 (37.0%)	97 (31.3%)	109 (24.0%)	
Saturated fatty acid, g/day	9.8 ± 5.4	11.4 ± 6.9	12.1 ± 7.6	<0.001	21.2 ± 16.4	17.4 ± 12.9	14.2 ± 11.7	<0.001
Total iron intake, mg/day	17.6 ± 8.0	20.2 ± 9.6	22.8 ± 10.7	<0.001	11.5 ± 9.5	12.2 ± 7.7	11.5 ± 7.1	0.406
Smoking status, n (%)				<0.001				<0.001
Current smoker	727 (64.5%)	466 (47.7%)	215 (26.3%)		168 (40.0%)	90 (29.0%)	50 (11.0%)	
Former smoker	294 (26.1%)	309 (31.6%)	261 (32.0%)		171 (40.7%)	119 (38.4%)	208 (45.7%)	
Never smoker	107 (9.5%)	202 (20.7%)	340 (41.7%)		81 (19.3%)	101 (32.6%)	197 (43.3%)	
Drinking status, n (%)				<0.001				<0.001
Heavy drinker	320 (28.4%)	188 (19.2%)	62 (7.6%)		101 (24.0%)	35 (11.3%)	32 (7.0%)	
Mild to moderate drinker	637 (56.5%)	524 (53.6%)	375 (46.0%)		247 (58.8%)	169 (54.5%)	200 (44.0%)	
Non-drinker	171 (15.2%)	265 (27.1%)	379 (46.4%)		72 (17.1%)	106 (34.2%)	223 (49.0%)	
Obesity status, n (%)				<0.001				<0.001
Obese	491 (43.5%)	240 (24.6%)	102 (12.5%)		273 (65.0%)	150 (48.4%)	108 (23.7%)	
Overweight	313 (27.7%)	323 (33.1%)	226 (27.7%)		87 (20.7%)	82 (26.5%)	132 (29.0%)	
Normal weight	324 (28.7%)	414 (42.4%)	488 (59.8%)		60 (14.3%)	78 (25.2%)	215 (47.3%)	
Abdominal obesity, n (%)	157 (13.9%)	55 (5.6%)	18 (2.2%)	<0.001	148 (35.2%)	73 (23.5%)	59 (13.0%)	<0.001
Omega-3/omega-6 PUFA ratio	0.139 ± 0.040	0.155 ± 0.048	0.171 ± 0.056	<0.001	0.158 ± 0.175	0.204 ± 0.165	0.240 ± 0.176	<0.001
Vitamin C intake, mg/day	80.3 ± 58.6	122.3 ± 87.5	161.2 ± 122.8	<0.001	51.1 ± 165.5	77.6 ± 132.5	95.5 ± 86.1	<0.001
Vitamin E intake, mg/day	11.7 ± 5.7	14.6 ± 7.3	17.4 ± 8.0	<0.001	7.1 ± 4.6	8.4 ± 4.4	8.7 ± 3.8	<0.001
Beta-carotene intake, μg/day	2564.1 ± 2134.9	3766.0 ± 3078.5	4905.9 ± 3687.3	<0.001	2183.1 ± 2501.2	3286.5 ± 3360.4	4350.8 ± 4891.9	<0.001
Physical activity, n (%)				<0.001				<0.001
Low (<7.5 METs-hr/day)	113 (10.0%)	39 (4.0%)	18 (2.2%)		242 (57.6%)	140 (45.2%)	153 (33.6%)	
Moderate (7.5–30 METs-hr/day)	720 (63.8%)	563 (57.6%)	428 (52.5%)		128 (30.5%)	122 (39.4%)	209 (45.9%)	
High (>30 METs-hr/day)	295 (26.2%)	375 (38.4%)	370 (45.3%)		50 (11.9%)	48 (15.5%)	93 (20.4%)	

* *p* value for the comparison of the baseline characteristics among sex-specific tertile groups of oxidative balance score at the baseline survey.

Significance was set at *p* < 0.05.

KoGES, Korean Genome and Epidemiology Study; KNHANES, Korean National Health and Nutrition Examination Survey; MBP, mean blood pressure; HDL, high-density lipoprotein; CRP, C-reactive protein; MetS, metabolic syndrome; PUFA, poly-unsaturated fatty acid; MET, metabolic equivalent of task.

In the KNHANES, the T3 men had older age and lower MBP, insulin, triglyceride, and WBC. The proportion of MetS was significantly higher in T1 men. The T3 men had lower intake of SFA, and higher intake of omega-3 to omega-6 PUFA ratio, vitamin C, vitamin E, and beta-carotene. The T3 men exhibited lower proportions of current smokers, heavy drinkers, obese, and abdominal obesity and greater high intensity physical activity in both the KoGES and KNHANES.

Table 3 shows the baseline characteristics of the women according to OBS tertile in the KoGES and KNHANES, respectively. In the KoGES, the T3 women had lower MBP, insulin, total cholesterol, triglyceride, and WBC. The proportion of participants with two MetS components was lowest in the T3 group. T3 women had higher intake of SFA, total iron, omega-3 to omega-6 PUFA ratio, vitamin C, vitamin E, and beta-carotene.

**Table 3:** Baseline characteristics of women in the KoGES and 2021 KNHANES.

	Oxidative balance score tertiles							
	Women in KoGES				Women in 2021KNHANES			
Variables	T1 (*n* = 828)	T2 (*n* = 1,027)	T3 (*n* = 1,031)	*p* ^*^	T1 (*n* = 611)	T2 (*n* = 481)	T3 (*n* = 458)	*p* ^*^
Age, years	51.6 ± 8.7	50.6 ± 8.6	49.1 ± 8.1	<0.001	47.7 ± 16.4	51.2 ± 16.3	54.8 ± 14.9	<0.001
MBP, mmHg	91.9 ± 12.5	91.3 ± 12.1	89.8 ± 12.1	<0.001	86.8 ± 11.0	86.0 ± 10.3	86.6 ± 11.4	0.478
Glucose, mg/dL	82.4 ± 12.0	81.3 ± 11.5	81.2 ± 13.0	0.061	98.9 ± 18.2	98.9 ± 18.5	98.5 ± 15.4	0.928
Insulin, IU/	7.9 ± 5.7	7.5 ± 4.0	7.2 ± 4.2	0.010	9.5 ± 6.2	8.8 ± 8.5	7.2 ± 5.0	<0.001
Total cholesterol, mg/dL	191.4 ± 35.0	187.2 ± 34.8	182.6 ± 32.1	<0.001	190.9 ± 37.4	189.0 ± 37.3	193.7 ± 38.3	0.165
Triglyceride, mg/dL	119.4 ± 49.0	117.1 ± 45.9	113.1 ± 51.2	0.017	112.2 ± 73.0	103.7 ± 67.6	97.0 ± 67.3	0.002
HDL cholesterol, mg/dL	48.2 ± 10.2	48.5 ± 10.3	48.6 ± 9.9	0.711	55.3 ± 12.9	56.2 ± 13.3	58.0 ± 13.5	0.005
WBC, 10^9^/μL	6.3 ± 1.8	6.2 ± 1.7	6.0 ± 1.7	0.001	5.9 ± 1.6	5.6 ± 1.5	5.4 ± 1.5	<0.001
Education level, n (%)				<0.001				0.462
Elementary/middle school	558 (67.7%)	640 (62.7%)	540 (52.6%)		147 (24.1%)	115 (23.9%)	111 (24.2%)	
High school	210 (25.5%)	316 (31.0%)	383 (37.3%)		152 (24.9%)	131 (27.2%)	136 (29.7%)	
College/university	56 (6.8%)	65 (6.4%)	104 (10.1%)		312 (51.1%)	235 (48.9%)	211 (46.1%)	
Household income, n (%)				0.001				0.358
<100 million Korean Won	297 (36.4%)	346 (34.2%)	307 (30.3%)		193 (31.6%)	151 (31.5%)	152 (33.3%)	
100–200 million Korean Won	256 (31.4%)	320 (31.7%)	292 (28.8%)		223 (36.6%)	152 (31.7%)	152 (33.3%)	
>200 million Korean Won	263 (32.2%)	345 (34.1%)	414 (40.9%)		194 (31.8%)	177 (36.9%)	153 (33.5%)	
Energy intake, kcal/day	1727.4 ± 544.0	1805.4 ± 629.3	2120.2 ± 848.0	<0.001	1563.7 ± 611.1	1578.7 ± 601.8	1589.8 ± 535.0	0.766
Number of MetS components, n (%)				<0.001				0.001
0	108 (13.0%)	214 (20.8%)	272 (26.4%)		209 (34.2%)	178 (37.0%)	152 (33.2%)	
1	296 (35.7%)	423 (41.2%)	452 (43.8%)		137 (22.4%)	100 (20.8%)	133 (29.0%)	
2	424 (51.2%)	390 (38.0%)	307 (29.8%)		100 (16.4%)	95 (19.8%)	88 (19.2%)	
≥3					165 (49.4%)	108 (22.4%)	85 (18.6%)	
Saturated fatty acid, g/day	9.3 ± 5.0	10.6 ± 6.3	12.9 ± 9.3	<0.001	15.2 ± 11.3	12.9 ± 9.8	10.3 ± 7.7	<0.001
Total iron intake, mg/day	15.6 ± 6.5	18.3 ± 8.9	23.1 ± 12.4	<0.001	8.7 ± 7.0	8.5 ± 5.0	9.4 ± 5.3	0.035
Smoking status, n (%)				<0.001				<0.001
Current smoker	49 (5.9%)	26 (2.5%)	6 (0.6%)		54 (8.8%)	7 (1.5%)	2 (0.4%)	
Former smoker	17 (2.1%)	9 (0.9%)	3 (0.3%)		49 (8.0%)	16 (3.3%)	9 (2.0%)	
Never smoker	762 (92.0%)	992 (96.6%)	1,022 (99.1%)		508 (83.1%)	458 (95.2%)	447 (97.6%)	
Drinking status, n (%)				<0.001				<0.001
Heavy drinker	28 (3.4%)	10 (1.0%)	5 (0.5%)		53 (8.7%)	11 (2.3%)	1 (0.2%)	
Mild to moderate drinker	327 (39.5%)	282 (27.5%)	187 (18.1%)		273 (44.7%)	167 (34.7%)	110 (24.0%)	
Non-drinker	473 (57.1%)	735 (71.6%)	839 (81.4%)		285 (46.6%)	303 (63.0%)	347 (75.8%)	
Obesity status, n (%)				<0.001				<0.001
Obese	452 (54.6%)	354 (34.5%)	138 (13.4%)		272 (44.5%)	119 (24.7%)	52 (11.4%)	
Overweight	234 (28.3%)	306 (29.8%)	295 (28.6%)		122 (20.0%)	121 (25.2%)	99 (21.6%)	
Normal weight	142 (17.1%)	367 (35.7%)	598 (58.0%)		217 (35.5%)	241 (50.1%)	307 (67.0%)	
Abdominal obesity, n (%)	259 (31.3%)	151 (14.7%)	57 (5.5%)	<0.001	99 (16.2%)	44 (9.1%)	8 (1.7%)	<0.001
Omega-3/omega-6 PUFA ratio	0.134 ± 0.037	0.154 ± 0.053	0.175 ± 0.067	<0.001	0.177 ± 0.190	0.239 ± 0.249	0.295 ± 0.268	<0.001
Vitamin C intake, mg/day	82.8 ± 65.4	123.6 ± 110.1	194.1 ± 147.0	<0.001	38.2 ± 46.4	66.1 ± 78.7	106.3 ± 114.9	<0.001
Vitamin E intake, mg/day	10.3 ± 5.7	13.1 ± 7.1	18.3 ± 10.9	<0.001	5.6 ± 3.2	6.4 ± 3.6	7.4 ± 3.4	<0.001
Beta-carotene intake, μg/day	2095.2 ± 1456.3	3144.4 ± 2730.2	5089.7 ± 4572.1	<0.001	1757.6 ± 1782.7	2690.9 ± 2388.5	4602.5 ± 4064.0	<0.001
Physical activity, n (%)				<0.001				<0.001
Low (<7.5 METs-hr/day)	127 (15.3%)	75 (7.3%)	53 (5.1%)		357 (58.4%)	221 (45.9%)	170 (37.1%)	
Moderate (7.5–30 METs-hr/day)	560 (67.6%)	702 (68.4%)	648 (62.9%)		213 (34.9%)	223 (46.4%)	218 (47.6%)	
High (>30 METs-hr/day)	141 (17.0%)	250 (24.3%)	330 (32.0%)		41 (6.7%)	37 (7.7%)	70 (15.3%)	

* *p* value for the comparison of the baseline characteristics among sex-specific tertile groups of oxidative balance score at the baseline survey.

Significance was set at *p* < 0.05.

KoGES, Korean Genome and Epidemiology Study; KNHANES, Korean National Health and Nutrition Examination Survey; MBP, mean blood pressure; HDL, high-density lipoprotein; CRP, C-reactive protein; MetS, metabolic syndrome; PUFA, poly-unsaturated fatty acid; MET, metabolic equivalent of task.

In the KNHANES, the T3 women had older age and higher HDL-cholesterol, but lower insulin, triglyceride, and WBC. The proportion of MetS was significantly higher in T1 women. The T3 women had lower intake of SFA and higher intake of omega-3 to omega-6 PUFA ratio, vitamin C, vitamin E, and beta-carotene. The T3 women had lower proportions of current smokers, heavy drinkers, obese, and abdominal obesity and greater high intensity physical activity in both the KoGES and KNHANES.

### 3.2. Longitudinal association of OBS with incident MetS

**Figure 2:**
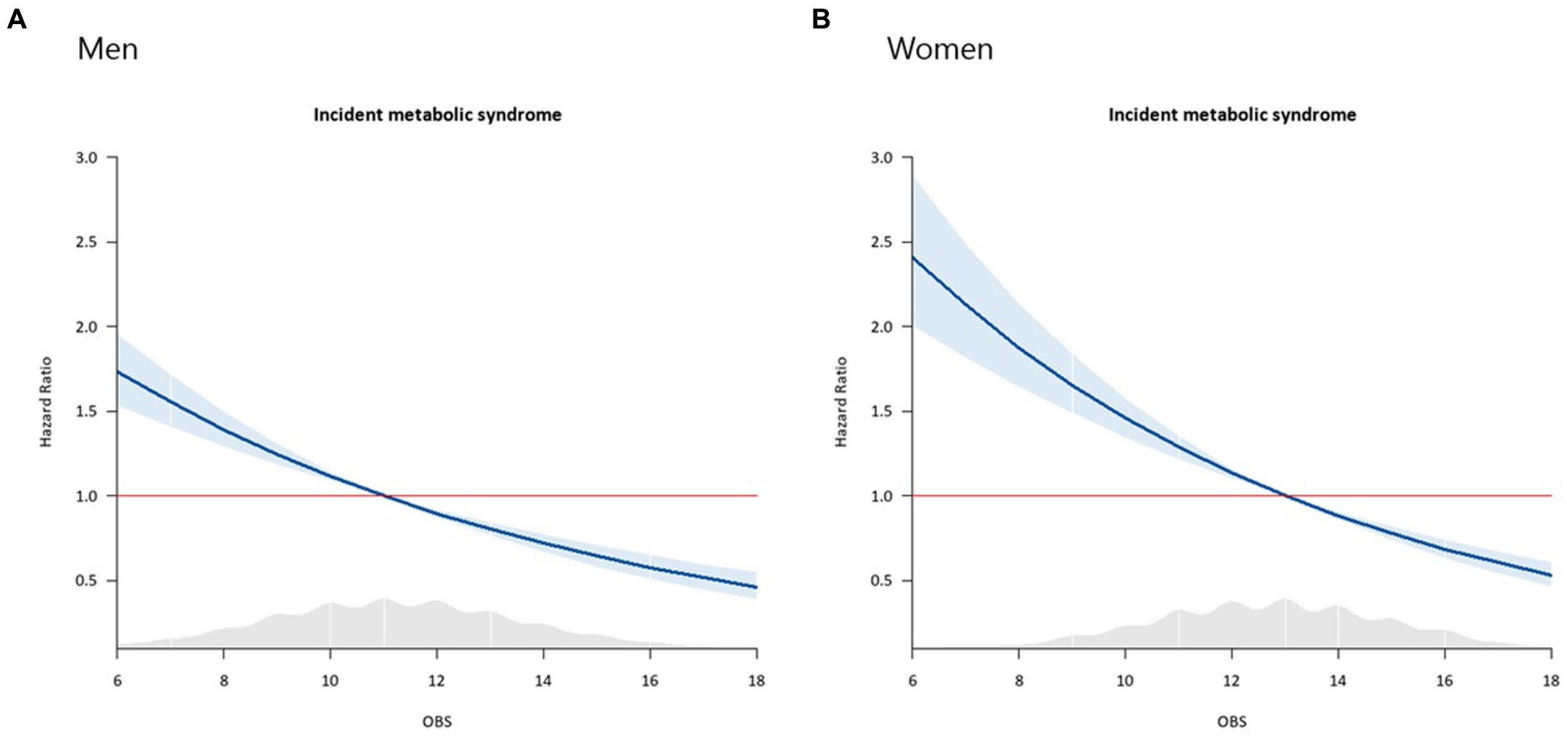
Cox proportional hazard spline curves showing an inverse relationship between oxidative balance scores and the risk of incident metabolic syndrome. The blue line illustrates changes in hazard ratios for the incidence of metabolic syndrome across a range of OBS values. The sky-blue area represents 95% confidence intervals. The red line indicates a hazard ratio of one. The gray shaded area denotes a density plot showing the distribution of the study population according to OBS values. **(A)** Men, **(B)** Women.

**Figure 3:**
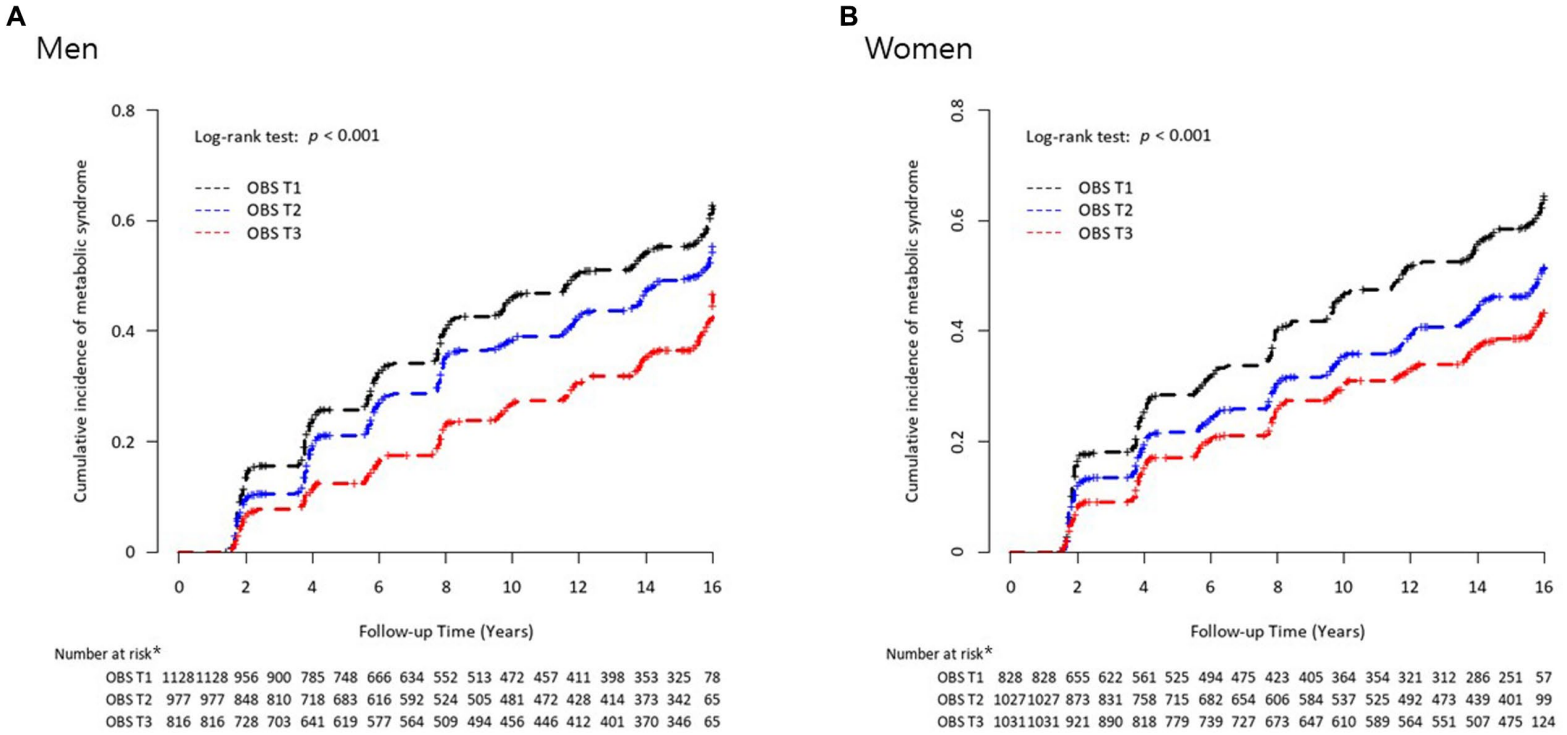
Kaplan–Meier curves of cumulative incidence rates of metabolic syndrome according to sex-specific oxidative balance score tertiles for **(A)** men and **(B)** women. ^*^Number at risk refers to those individuals at each time point who are still under observation and have not yet experienced metabolic syndrome.

During the mean 13.6-year follow-up period, 1,296 (44.4%) men and 880 (30.5%) women developed new-onset MetS in the KoGES. Baseline characteristics of men and women from KoGES are shown in Supplementary Table 3. Inverse relationships were noted between OBS and the risk of incident MetS in both men (Figure 2A) and women (Figure 2B). Figure 3 presents the cumulative incidence rates of MetS as Kaplan–Meier curves according to sex-specific OBS tertiles in the KoGES. The T3 group showed the lowest cumulative incidence of MetS, followed by the T2 and T1 groups, among both men (Figure 3A) and women (Figure 3B) (both log-rank test *p*-values <0.001).

Table 4 shows the independent association between OBS tertiles and MetS in the KoGES and the 2021 KNHANES. In the KoGES, the HRs (95% CIs) for new-onset MetS per increment in OBS were 0.90 (0.88–0.92) in men and 0.88 (0.86–0.91) in women. Compared to referent T1, the adjusted HRs (95% CIs) for new-onset MetS were 0.82 (0.72–0.93) in T2 and 0.56 (0.48–0.62) in T3 among men. Compared to referent T1, the adjusted HRs (95% CIs) for new-onset MetS were 0.71 (0.62–0.81) in T2 and 0.63 (0.55–0.73) in T3 among women.

**Table 4:** Associations between oxidative balance score and incident/prevalent metabolic syndrome in KoGES and KNHANES.

KoGES								
Oxidative balance score tertiles	Numbers, n	New-onset MetS cases, n	Follow-up period, person-year	Incidence rate per 1,000 person-years	Unadjusted		Adjusted	
					HR (95% CI)	*p*	HR (95% CI)	*p*
Men								
Continuous (per increment)					0.90 (0.88–0.92)	<0.001	0.90 (0.88–0.92)	<0.001
T1	1,128	582	10028.0	58.0	1		1	
T2	977	440	9414.4	46.7	0.81 (0.72–0.92)	<0.001	0.82 (0.72–0.93)	0.002
T3	816	274	8610.4	31.8	0.56 (0.48–0.64)	<0.001	0.56 (0.48–0.65)	<0.001
Women								
Continuous (per increment)					0.88 (0.86–0.91)		0.90 (0.88–0.93)	<0.001
T1	828	452	7420.6	60.9	1		1	
T2	1,027	443	10264.1	43.2	0.71 (0.62–0.81)	<0.001	0.71 (0.62–0.81)	<0.001
T3	1,031	388	11217.2	34.6	0.57 (0.50–0.65)	<0.001	0.63 (0.55–0.73)	<0.001
KNHANES								
Oxidative balance score tertiles	Numbers, n	MetS cases, n		Prevalence rate of MetS, %	Unadjusted		Adjusted	
					OR (95% CI)	*p*	OR (95% CI)	*p*
Men								
Continuous (per increment)					0.89 (0.85–0.93)	<0.001	0.87 (0.81–0.92)	<0.001
T1	420	155		36.90	1		1	
T2	310	97		31.29	0.78 (0.57–1.06)	0.115	0.67 (0.45–1.00)	0.051
T3	455	109		23.96	0.54 (0.40–0.72)	<0.001	0.44 (0.29–0.65)	<0.001
Women								
Continuous (per increment)					0.88 (0.84–0.93)		0.80 (0.75–0.86)	<0.001
T1	611	165		27.00	1		1	
T2	481	108		22.45	0.59 (0.59–1.03)	0.085	0.58 (0.40–0.86)	0.006
T3	458	85		18.56	0.62 (0.46–0.83)	0.001	0.34 (0.23–0.50)	<0.001

Adjusted for age, education level, monthly household income, total energy intake, mean blood pressure, whole blood white blood cell count, fasting plasma glucose, and serum total cholesterol levels based on characteristics for each population. Significance was set at *p* < 0.05.

KoGES, Korean Genome and Epidemiology Study; KNHANES, Korean National Health and Nutrition Examination Survey; MetS, metabolic syndrome; HR, hazard ratio; CI, confidence interval.

In the 2021 KNHANES, the ORs (95% CIs) for MetS per increment in OBS were 0.89 (0.85–0.93) in men and 0.88 (0.84–0.93) in women. Compared to referent T1, the adjusted ORs (95% CIs) for MetS in T3 were 0.44 (0.29–0.65) in men and 0.34 (0.23–0.50) in women. When we conducted the subgroup analysis in adults aged 40–69 years from KNHANES, the significant association between OBS and incident MetS remained (T3 vs. T1, adjusted ORs and 95% CIs = 0.39 [0.24–0.65] in men and 0.26 [0.16–0.43] in women) (Supplementary Table 4).

## 4. Discussion

We found that OBS was independently and inversely related to the prevalence of MetS and incident MetS in men and women separately after adjusting for potential confounders in both cross-sectional and longitudinal datasets.

Higher OBS values were closely associated with a lower risk of incident MetS. Our findings are line with previous studies that have demonstrated the association with chronic diseases such hypertension (37), non-alcoholic fatty liver diseases (NAFLD) (38), cardiovascular diseases (39) and MetS (18). While limited studies have reported on the link between OBS and MetS in Caucasian populations, Annor et al. found an inverse association between OBS and hypertension, a component of MetS, in a racially diverse population (37). A cross-sectional study conducted in Korea found that higher OBS was associated with a lower risk of MetS (18). The study further revealed that individuals in the highest OBS quartile exhibited lower levels of inflammatory markers, including white blood cell count and C-reactive protein. A recent study has revealed that OBS exhibits an inverse association with NAFLD, a condition sharing a similar spectrum with metabolic syndrome (38).

In contrast to our current study, Noruzi et al. (19) reported an inverse association between higher OBS values and a reduced likelihood of MetS components, such as abdominal obesity and elevated DBP, among an Iranian population. However, their study did not find a significant association between OBS and the overall prevalence of MetS or other individual MetS components. Several factors could explain the disparities observed in the previous study. One contributing factor is that the previous Iranian study did not include comprehensive data on alcohol consumption, which is an important component of OBS. Additionally, differences in demographic factors, including age, ethnicity, and the composition of participant samples, could contribute to the observed differences. Furthermore, variations in the composition of OBS components and discrepancies in sample sizes may also contribute to these disparities.

Many studies have suggested various OBS values with different scoring schemes and with different types of anti-and pro-oxidant components, including dietary factors and lifestyle factors (14). Similar to previous studies, we aimed to construct an OBS using OBS components available in the KoGES and KNHANES as much as possible. Also, we found that higher scores for the OBS used in the current study were correlated with lower blood glucose, insulin, total cholesterol, triglyceride, and CRP.

There are several plausible explanations for the findings of this study. First, a higher OBS indicates a favorable antioxidant defense system, which can counteract excessive oxidative stress, a key contributor to MetS development (10). Previous studies have indicated that healthy lifestyles that include aerobic exercise, high consumption of vegetables, alcohol restriction, and body weight control are associated with decreased blood pressure, insulin resistance, and other related diseases by controlling oxidative stress (40, 41). Although the highest tertile group in KoGES showed the highest SFA and total iron intake, compared with the other tertile groups, this could be due to the tertile group having the highest total energy intake. However, considering that the relationship between OBS and incident MetS remained significant even after adjusting for total energy intake, the effect of antioxidants and lifestyle factors could outweigh that of the pro-oxidant components. Second, chronic systemic inflammation can contribute to the development of MetS. Chronic inflammation may worsen insulin action, increase blood pressure, and deteriorate lipid metabolism (42, 43). A randomized controlled trial found that participants in the highest quartile of white blood cell counts had a higher probability of having MetS than those in the lowest baseline sex-adjusted quartile (OR: 2.47, 95% CI: 2.03–2.99, p for trend <0.001) (44). Adopting a healthy dietary pattern characterized by a high intake of vegetables, nuts and fish, quitting smoking, and maintaining a healthy body weight are recognized as effective measures for mitigating chronic inflammation (45). We found that OBS was negatively correlated with CRP in the current study. Third, elevated OBS values have the potential to enhance insulin sensitivity, facilitate efficient glucose utilization, and reduce the risk of insulin resistance, which is a crucial factor in the development of MetS. Physical activity improves peripheral insulin sensitivity (46), while smoking can have detrimental effects on pancreatic β-cell function and insulin sensitivity (47).

Finally, the metabolic overburden of mitochondria, which causes incomplete β-oxidation and an accumulation of lipotoxicity, is a major contributor to both β-cell dysfunction and muscle insulin resistance (48). Moreover, a genome-wide association study revealed that the VEGF signaling pathway, glutathione metabolism, and the Rac-1 pathway were highly enhanced biological pathways associated with OBS and MetS (18). Follow-up studies are needed to determine differences in genetic variations, such as single nucleotide polymorphisms, according to OBS values.

This study has some limitations. First, there were differences in sociodemographic factors and some items for the OBS between individuals included in the analysis and those excluded from the analysis (Supplementary Tables 5, 6). Thus, there is a possibility of selection bias. However, we analyzed the KOGES and KNHANES datasets separately and observed significant results in each analysis. We also found significant associations between OBS and MetS in adults who aged 40–69 years in the KNHANES. Second, in the analysis of the KoGES dataset, only OBS values from the baseline survey were considered due to the unavailability of follow-up information specifically related to diet. All variables included in the OBS can change over time. Therefore, in future studies, the effect of changes in OBS over time on the incidence of MetS should be analyzed. Third, information about dietary components was obtained from FFQs. Although FFQs are useful for investigating nutritional status in large-scale epidemiology studies, they lack accuracy on absolute nutrient values, especially micronutrients, and may over- or under-report consumption of certain foods (49). Due to a lack of information, we could not consider blood micronutrient levels. In addition, there is a possibility of recall bias in the information on average intake over the past year. Fourth, each component included in the OBS may have distinct effects on MetS. It is important to consider an analysis method that takes into account the weights associated with each pro-oxidant and anti-oxidant component when assessing their impact on incident MetS. Further controlled clinical trials considering more detailed anti-and pro-oxidants components should be performed to confirm a causal relationship between OBS and incident MetS. Also, efforts are needed to construct a verified OBS. Finally, we could not consider the impact of all possible pro- and anti-inflammatory cytokines, including interleukin (IL)-1β, IL-4, IL-6, IL-10, and tumor necrosis factor-α.

Despite these limitations, we are the first to report a significant association between OBS and MetS in a large, population-based, prospective study with a long follow-up period. The present study found higher OBS values were significantly associated with lower incidences of MetS in two independent large population-based datasets. OBS-enhancing strategies, including maintaining a healthy weight, engaging in regular exercise, quitting smoking, and consuming antioxidant substances, may successfully reduce one’s risk of developing MetS. It is imperative to conduct more randomized controlled studies to confirm the validity of authorized OBS diet recommendations for preventing MetS.

## Data availability statement

Publicly available datasets were analyzed in this study. This data can be found here: the Korean Genome and Epidemiology Study data are available through a procedure described at: https://nih.go.kr/ko/main/main.do. The Korean National Health and Nutritional Examination Survey data are available through a procedure described at: https://knhanes.kdca.go.kr/knhanes/sub03/sub03_02_05.do.

## Ethics statement

The studies involving humans were approved by IRB of Yongin Severance Hospital. The studies were conducted in accordance with the local legislation and institutional requirements. The participants provided their written informed consent to participate in this study.

## Author contributions

H-MP, T-HH, J-HL, and Y-JK: study concept and design, acquisition, analysis, interpretation of data, drafting the manuscript, and approval of the final manuscript and had the final responsibility to submit the study for publication. J-HL and Y-JK: study concept and design, interpretation of data, supervision, and revising the manuscript. All authors contributed to the article and approved the submitted version.

## Funding

This research was financially supported by the Ministry of SMEs and Startups and Korea Technology and Promotion Agency for SMEs (TIPA) through the Regional Specialized Industry Development Plus Program (Grant number: S3370378).

## Conflict of interest

The authors declare that the research was conducted in the absence of any commercial or financial relationships that could be construed as a potential conflict of interest.

## Publisher’s note

All claims expressed in this article are solely those of the authors and do not necessarily represent those of their affiliated organizations, or those of the publisher, the editors and the reviewers. Any product that may be evaluated in this article, or claim that may be made by its manufacturer, is not guaranteed or endorsed by the publisher.
