# Supplementary material for: Oxidative balance score inversely associated with the prevalence and incidence of metabolic syndrome: analysis of two studies of the Korean population
Source: Front Nutr. 2023 Aug 16;10:1226107. doi: 10.3389/fnut.2023.1226107 (PMC10466805; doi:10.3389/fnut.2023.1226107)
Supplement: Supplementary file 1 [file Table_1.docx]

Supplementary Material

Oxidative balance score is inversely associated with prevalence and incidence of metabolic syndrome: Analysis of two studies of the Korean population

Hye-Min Park^1^, Tea-Hwa Han^2^, Yu-Jin Kwon^3^*, and Jun-Hyuk Lee^4,5^*

*** Correspondence:** Yu-Jin Kwon MD, PhD; [digda3@yuhs.ac](mailto:digda3@yuhs.ac)

Jun-Hyuk Lee MD; [swpapa@eulji.ac.kr](mailto:swpapa@eulji.ac.kr)

**Supplementary Table 1.** Results of collinearity test in men

|  | Men in the KoGES | | Men in the KNHANES | |
| --- | --- | --- | --- | --- |
|  | VIF |  | VIF |  |
| Age | 1.46 |  | 1.54 |  |
| MBP | 1.06 |  | 1.10 |  |
| Glucose | 1.03 |  | 1.12 |  |
| Total cholesterol | 1.12 |  | 1.07 |  |
| WBC | 1.03 |  | 1.02 |  |
| Education level | 1.32 |  | 1.54 |  |
| Household income | 1.52 |  | 1.20 |  |
| Energy intake | 1.04 |  | 1.06 |  |

Abbreviations: KoGES, Korean Genome and Epidemiology Study; KNHANES, Korean National Health and Nutrition Examination Survey, VIF, variance of inflation factor, MBP, mean blood pressure; WBC, white blood cell.

**Supplementary Table 2.** Results of collinearity test in women

|  | Women in the KoGES | | Women in the KNHANES | |
| --- | --- | --- | --- | --- |
|  | VIF |  | VIF |  |
| Age | 1.52 |  | 2.10 |  |
| MBP | 1.17 |  | 1.21 |  |
| Glucose | 1.02 |  | 1.15 |  |
| Total cholesterol | 1.12 |  | 1.06 |  |
| WBC | 1.01 |  | 1.06 |  |
| Education level | 1.33 |  | 1.99 |  |
| Household income | 1.43 |  | 1.25 |  |
| Energy intake | 1.06 |  | 1.02 |  |

Abbreviations: KoGES, Korean Genome and Epidemiology Study; KNHANES, Korean National Health and Nutrition Examination Survey, VIF, variance of inflation factor, MBP, mean blood pressure; WBC, white blood cell.

**Supplementary Table 3.** Baseline characteristics of men and women in Korean Genome and Epidemiology Study

| Variables | Men | Women | Total | p |
| --- | --- | --- | --- | --- |
|  | (*n* = 2921) | (*n* = 2886) | (*n* = 5807) |  |
| Age, years | 51.4 ± 8.8 | 50.4 ± 8.5 | 50.9 ± 8.7 | < 0.001 |
| MBP, mmHg | 95.4 ± 12.0 | 90.9 ± 12.2 | 93.2 ± 12.3 | < 0.001 |
| Glucose, mg/dL | 86.3 ± 15.9 | 81.6 ± 12.2 | 83.9 ± 14.4 | < 0.001 |
| Insulin, IU/ | 6.5 ± 3.5 | 7.5 ± 4.6 | 7.0 ± 4.1 | < 0.001 |
| Total cholesterol, mg/dL | 190.4 ± 35.1 | 186.8 ± 34.1 | 188.6 ± 34.7 | < 0.001 |
| Triglyceride, mg/dL | 148.8 ± 95.2 | 116.3 ± 48.8 | 132.7 ± 77.5 | < 0.001 |
| HDL cholesterol, mg/dL | 45.8 ± 9.9 | 48.4 ± 10.1 | 47.1 ± 10.1 | < 0.001 |
| WBC, 10^9^/μL | 6.7 ± 1.8 | 6.1 ± 1.7 | 6.4 ± 1.8 | < 0.001 |
| Education level, n (%) |  | | | < 0.001 |
| Elementary/middle school | 1201 (41.3%) | 1738 (60.5%) | 2939 (50.8%) |  |
| High school | 1062 (36.5%) | 909 (31.7%) | 1971 (34.1%) |  |
| College/university | 648 (22.3%) | 225 (7.8%) | 873 (15.1%) |  |
| Household income, n (%) |  | | | < 0.001 |
| < 100 million Korean Won | 788 (27.1%) | 950 (33.5%) | 1738 (30.3%) |  |
| 100–200 million Korean Won | 880 (30.3%) | 868 (30.6%) | 1748 (30.4%) |  |
| > 200 million Korean Won | 1235 (42.5%) | 1022 (36.0%) | 2257 (39.3%) |  |
| Energy intake, kcal/day | 2020.6 ± 678.7 | 1895.5 ± 715.2 | 1958.4 ± 699.8 | < 0.001 |
| Number of MetS components, n (%) |  | | | 0.001 |
| 0 | 718 (24.6%) | 594 (20.6%) | 1312 (22.6%) |  |
| 1 | 1123 (38.4%) | 1171 (40.6%) | 2294 (39.5%) |  |
| 2 | 1080 (37.0%) | 1121 (38.8%) | 2201 (37.9%) |  |
| Saturated fatty acid, g/day | 11.0 ± 6.7 | 11.0 ± 7.4 | 11.0 ± 7.0 | 0.704 |
| Total iron intake, mg/day | 19.9 ± 9.6 | 19.2 ± 10.3 | 19.6 ± 9.9 | 0.010 |
| Smoking status, n (%) |  | | | < 0.001 |
| Current smoker | 1408 (48.2%) | 81 (2.8%) | 1489 (25.6%) |  |
| Former smoker | 864 (29.6%) | 29 (1.0%) | 893 (15.4%) |  |
| Never smoker | 649 (22.2%) | 2776 (96.2%) | 3425 (59.0%) |  |
| Drinking status, n (%) |  | | | < 0.001 |
| Heavy drinker | 570 (19.5%) | 43 (1.5%) | 613 (10.6%) |  |
| Mild to moderate drinker | 1536 (52.6%) | 796 (27.6%) | 2332 (40.2%) |  |
| Non-drinker | 815 (27.9%) | 2047 (70.9%) | 2862 (49.3%) |  |
| Obesity status, n (%) |  | | | 0.001 |
| Obese | 833 (28.5%) | 944 (32.7%) | 1777 (30.6%) |  |
| Overweight | 862 (29.5%) | 835 (28.9%) | 1697 (29.2%) |  |
| Normal weight | 1226 (42.0%) | 1107 (38.4%) | 2333 (40.2%) |  |
| Abdominal obesity, n (%) | 230 (7.9%) | 467 (16.2%) | 697 (12.0%) | < 0.001 |
| omega-3/omega-6 PUFA ratio | 0.2 ± 0.0 | 0.2 ± 0.1 | 0.2 ± 0.1 | 0.105 |
| Vitamin C intake, mg/day | 116.9 ± 95.8 | 137.1 ± 123.8 | 126.9 ± 111.0 | < 0.001 |
| Vitamin E intake, mg/day | 14.3 ± 7.3 | 14.2 ± 9.0 | 14.2 ± 8.2 | 0.694 |
| Beta-carotene intake, μg/day | 3620.3 ± 3101.9 | 3538.3 ± 3497.9 | 3579.6 ± 3304.6 | 0.345 |
| Physical activity, n (%) |  | | | < 0.001 |
| Low (<7.5 METs-hr/day) | 170 (5.8%) | 255 (8.8%) | 425 (7.3%) |  |
| Moderate (7.5–30 METs-hr/day) | 1711 (58.6%) | 1910 (66.2%) | 3621 (62.4%) |  |
| High (>30 METs-hr/day) | 1040 (35.6%) | 721 (25.0%) | 1761 (30.3%) |  |
| Mean OBS | 11.2 ± 2.2 | 12.7 ± 2.1 | 11.9 ± 2.3 | < 0.001 |

*^*^p* value for the comparison of the baseline characteristics between men and women.

Significance was set at *p* < 0.05.

**Supplementary Table 4**. Multiple logistic regression analysis showing the relationship of oxidative balance scores with prevalent metabolic syndrome in adults aged 40-69years using data from 2021 Korean Nutrition and Health Examination Survey.

| Oxidative balance score tertiles |  | Numbers, n | MetS cases, n | Prevalence rate of MetS, % | Unadjusted |  | Adjusted |  |
| --- | --- | --- | --- | --- | --- | --- | --- | --- |
|  |  |  |  |  | OR (95% CI) | *p* | OR (95% CI) | *p* |
| Men |  |  |  |  |  |  |  |  |
| Continuous (per increment) |  |  |  |  | 0.82  (0.77–0.87) | <0.001 | 0.84  (0.78–0.91) | <0.001 |
| T1 |  | 212 | 103 | 48.6 | 1 (reference) |  | 1 (reference) |  |
| T2 |  | 180 | 70 | 38.9 | 0.67  (0.45–1.01) | 0.054 | 0.74  (0.46–1.18) | 0.206 |
| T3 |  | 245 | 60 | 24.5 | 0.34  (0.23–0.51) | <0.001 | 0.39  (0.24–0.65) | <0.001 |
| Women |  |  |  |  |  |  |  |  |
| Continuous (per increment) |  |  |  |  | 0.82  (0.77–0.87) | <0.001 | 0.78  (0.72–0.85) | <0.001 |
| T1 |  | 330 | 115 | 34.8 | 1 (reference) |  | 1 (reference) |  |
| T2 |  | 287 | 66 | 23.0 | 0.56  (0.39–0.80) | 0.001 | 0.55  (0.35–0.87) | 0.011 |
| T3 |  | 309 | 47 | 15.2 | 0.34  (0.29–0.49) | <0.001 | 0.26  (0.16–0.43) | <0.001 |

Adjusted for age, education level, monthly household income, total energy intake, mean blood pressure, whole blood white blood cell count, fasting plasma glucose, and serum total cholesterol levels.

Significance was set at *p* < 0.05. Abbreviations: MetS, metabolic syndrome; OR, odds ratio; CI, confidence interval.

**Supplementary Table 5.** Baseline characteristics of the population for individuals included and excluded from the analysis

| Variables | Excluded | Included | Total | *p** |
| --- | --- | --- | --- | --- |
|  | (*n* = 4223) | (*n* = 5807) | (*n* = 10,030) |  |
| Male sex, n (%) | 1837 (43.5%) | 2921 (50.3%) | 4758 (47.4%) | <0.001 |
| Age, years | 54.2 ± 8.9 | 50.9 ± 8.7 | 52.3 ± 8.9 | <0.001 |
| MBP, mmHg | 101.8 ± 13.0 | 93.2 ± 12.3 | 96.8 ± 13.3 | <0.001 |
| Glucose, mg/dL | 92.2 ± 27.9 | 83.9 ± 14.4 | 87.3 ± 21.4 | <0.001 |
| Insulin, IU/ | 8.6 ± 5.6 | 7.0 ± 4.1 | 7.7 ± 4.8 | <0.001 |
| Total cholesterol, mg/dL | 194.7 ± 37.1 | 188.6 ± 34.7 | 191.2 ± 35.8 | <0.001 |
| Triglyceride, mg/dL | 203.5 ± 122.5 | 132.7 ± 77.5 | 162.5 ± 105.0 | <0.001 |
| HDL cholesterol, mg/dL | 41.2 ± 9.0 | 47.1 ± 10.1 | 44.6 ± 10.1 | <0.001 |
| WBC, 109/μL | 6.8 ± 1.8 | 6.4 ± 1.8 | 6.6 ± 1.8 | <0.001 |
| Education level, n (%) |  | | | <0.001 |
| Elementary/middle school | 2699 (64.8%) | 2939 (50.8%) | 5638 (56.7%) |  |
| High school | 1033 (24.8%) | 1971 (34.1%) | 3004 (30.2%) |  |
| College/university | 432 (10.4%) | 873 (15.1%) | 1305 (13.1%) |  |
| Household income, n (%) |  | | | <0.001 |
| <100 million Korean Won | 1783 (43.4%) | 1738 (30.3%) | 3521 (35.8%) |  |
| 100–200 million Korean Won | 1144 (27.9%) | 1748 (30.4%) | 2892 (29.4%) |  |
| > 200 million Korean Won | 1177 (28.7%) | 2257 (39.3%) | 3434 (34.9%) |  |
| Energy intake, kcal/day | 1954.9 ± 735.0 | 1958.4 ± 699.8 | 1957.0 ± 714.1 | 0.812 |
| Number of metabolic syndrome components, n (%) |  | | | <0.001 |
| 0 | 236 (5.6%) | 1312 (22.6%) | 1548 (15.4%) |  |
| 1 | 414 (9.8%) | 2294 (39.5%) | 2708 (27.0%) |  |
| 2 | 376 (8.9%) | 2201 (37.9%) | 2577 (25.7%) |  |
| 3 | 1894 (44.8%) | 0 (0.0%) | 1894 (18.9%) |  |
| 4 | 1066 (25.2%) | 0 (0.0%) | 1066 (10.6%) |  |
| 5 | 237 (5.6%) | 0 (0.0%) | 237 (2.4%) |  |

*^*^*The p-value is provided for the comparison of baseline characteristics between individuals who were excluded and included in the analysis. Significance was set at *p* <0.05. Abbreviations: MBP, mean blood pressure; HDL, high-density lipoprotein; CRP, C-reactive protein.

**Supplementary Table 6.** Individual components of the oxidative balance score for individuals included and excluded from the analysis

| Variables | Excluded | Included | Total | *p** |
| --- | --- | --- | --- | --- |
|  | (*n* = 4223) | (*n* = 5807) | (*n* = 10,030) |  |
| Saturated fatty acid, g/day | 10.6 ± 7.2 | 11.0 ± 7.0 | 10.9 ± 7.1 | 0.007 |
| Total iron intake, mg/day | 19.9 ± 10.4 | 19.6 ± 9.9 | 19.7 ± 10.1 | 0.136 |
| Smoking status, n (%) |  | | | 0.001 |
| Current smoker | 946 (23.1%) | 1489 (25.6%) | 2435 (24.6%) |  |
| Former smoker | 578 (14.1%) | 893 (15.4%) | 1471 (14.9%) |  |
| Never smoker | 2565 (62.7%) | 3425 (59.0%) | 5990 (60.5%) |  |
| Drinking status, n (%) |  | | | <0.001 |
| Heavy drinker | 406 (9.8%) | 613 (10.6%) | 1019 (10.3%) |  |
| Mild to moderate drinker | 1340 (32.4%) | 2332 (40.2%) | 3672 (36.9%) |  |
| Non-drinker | 2386 (57.7%) | 2862 (49.3%) | 5248 (52.8%) |  |
| Obesity status, n (%) |  | | | <0.001 |
| Obese | 2513 (59.6%) | 1777 (30.6%) | 4290 (42.8%) |  |
| Overweight | 911 (21.6%) | 1697 (29.2%) | 2608 (26.0%) |  |
| Normal weight | 795 (18.8%) | 2333 (40.2%) | 3128 (31.2%) |  |
| Abdominal obesity, n (%) | 2342 (55.6%) | 697 (12.0%) | 3039 (30.3%) | <0.001 |
| omega-3/omega-6 PUFA ratio | 0.2 ± 0.1 | 0.2 ± 0.1 | 0.2 ± 0.1 | 0.017 |
| Vitamin C intake, mg/day | 134.1 ± 114.7 | 126.9 ± 111.0 | 129.8 ± 112.6 | 0.002 |
| Vitamin E intake, mg/day | 13.9 ± 8.3 | 14.2 ± 8.2 | 14.1 ± 8.2 | 0.085 |
| Beta-carotene intake, μg/day | 3659.8 ± 3631.2 | 3579.6 ± 3304.6 | 3611.8 ± 3439.5 | 0.269 |
| Physical activity, n (%) |  | | | <0.001 |
| Low (<7.5 METs-hr/day) | 367 (9.6%) | 425 (7.3%) | 792 (8.2%) |  |
| Moderate (7.5–30 METs-hr/day) | 2276 (59.5%) | 3621 (62.4%) | 5897 (61.2%) |  |
| High (>30 METs-hr/day) | 1182 (30.9%) | 1761 (30.3%) | 2943 (30.6%) |  |

*^*^*The p-value is provided for the comparison of individual components for the oxidative balance score between individuals who were excluded and included in the analysis. Significance was set at *p* <0.05. Abbreviations: PUFA, poly-unsaturated fatty acid; MET, metabolic equivalent of task.
